# Health Status of Workers Engaged in the Small-scale Garment Industry: How Healthy are They?

**DOI:** 10.4103/0970-0218.62584

**Published:** 2010-01

**Authors:** Tushar Kanti Saha, Aparajita Dasgupta, Arindam Butt, Onkarnath Chattopadhyay

**Affiliations:** Department of PSM, A.I.I.H & P.H, Kolkata - 73, India

## Introduction

Though India is now considered a major power and is turning into a developed country from a developing country, a large section of its population still belong to the poorest of the poor. In developing countries, great efforts are directed towards the advancement of small-scale industries as these are considered the engine for their economic growth. According to WHO, over 1000 million people worldwide are employed in small-scale industries.(1) The ‘garment’ industry of India is one such industry. It is an unorganized sector, mostly run by private establishments. It provides employment for both men and women, mainly those from the lower socioeconomic classes.

The employees of this industry hardly ever benefit from occupational health-and-safety provisions. As a result their health suffers.(1) Studies show that musculoskeletal problems, diseases of the respiratory system and eye, accidents, injuries, skin diseases, stress, insomnia, etc. are all common among these workers. The ill health is compounded by various socioeconomic factors such as poverty, lack of education, poor working conditions, excess working hours, and poor diet.(2–4)

Against this background, and because no such study has been carried out in this part of the country to date, we conducted this study among workers employed in the ‘garment’ industry; we had the following objectives:

1. To find out the morbidity profile of the workers with special reference to musculoskeletal disorders.
2. To assess the relationship of socio-demographic and occupational factors with the musculoskeletal disorders.
3. To assess the felt needs of these workers.

## Materials and Methods

This was a community-based cross-sectional study carried out in a slum area of Kolkata. The study was conducted from September 2008 - November 2008.

There are three main areas in the slum where almost all those engaged in the small-scale garment industry reside. One such area was chosen by simple random sampling. A complete enumeration of all workers in the chosen area was done. There were one hundred and twelve such workers who were identified and all of them consented to participate in the study. The subjects were then interviewed using a predesigned, pre-tested, semi structured questionnaire that had been translated into the local language; the questionnaire collected data on socioeconomic conditions, occupational history, health problems.

The felt needs of the workers were obtained from two Focus Group Discussions in each of which 9 workers participated. The researchers organized the group discussions after assuring confidentiality to the participants. Efforts were made to elicit the problems faced by them at their place of work, dealings with their employer and the changes they would like for a better and conducive working environment.

## Results

The sociodemographic profile of the study population shows that most of the workers were males (76.79%) and were in the 15-45 age-group (80.36%), with none below 14 years of age [Table 1]. We found that 23.21% of the workers were illiterate and most of them belonged to poor socioeconomic status.

**Table 1:** Occupational and sociodemographic correlates of musculoskeletal morbidity among the wokers in the garment industry (*n* = 112)

Occupational risk factors	Total workers (%)	Musculoskeletal symptoms present (%)	Statistical significant
Years of working			Significant; χ^2^ = 16.94; *P* = 0.0002, df = 2
<5	18 (16.07)	6 (33.33)	
5-10	34 (30.36)	22 (64.71)	
>10	60 (53.57)	50 (83.33)	
Hours of work per day			Significant; χ^2^ = 12.67; *P* = 0.0018, df = 2
<5	16 (14.29)	6 (37.50)	
5-10	32(28.57)	20 (62.50)	
>10	64 (57.14)	52 (81.25)	
Nature of work			Significant; χ^2^ = 6.38; *P* = 0.0412 df = 2
Sewing	68 (60.71)	52 (76.47)	
Cutting	30 (26.79)	20 (66.67)	
Delivery	14 (12.50)	6 (42.86)	
Sociodemographic factors			
Age (years)			OR = 0.62 (NS); χ^2^ = 0.85; *P* = 0.3853, df = 1
15-45	90 (80.36)	61 (67.78)	
>45	22 (19.64)	17 (77.27)	
Sex			OR = 0.62 (NS); χ^2^ = 0.85; *P* = 0.3569, df= 1
Male	86 (76.79)	58 (67.44)	
Female	26 (23.21)	20 (76.93)	
Educational status			OR = 1.24 (NS); χ^2^ = 0.19; *P* = 0.6639, df = 1
Illiterate	26 (23.21)	19 (73.08)	
Literate	86 (76.79)	59 (68.60)	
Addiction			OR =0.53 (NS); χ^2^ = 2.26; *P* = 0.1327, df = 1
Present	74 (66.07)	55 (74.32)	
Absent	38 (33.93)	23 (60.53)	
Per capital income (Rs.)			OR = 1.07 (NS); χ^2^ = 0.02; *P* = 0.8862, df = 1
<500	24 (21.43)	17 (70.83)	
>500	88 (78.57)	61 (92.32)	

The average monthly per capita income was Rs. 500-1000. Addiction was rampant that is more than two-thirds (66.07%) of the workers were addicted to one or more substances i.e. tobacco (37.50%), alcohol (12.50%) and both (16.07%).

On enquiring about their chief complaints we found that musculoskeletal problems (69.64%) were the commonest health problem. The body areas commonly affected was neck (64.10%), low back (41.03%), hand, wrist, finger, and shoulder. The common symptoms in these subjects were pain (69.23%), weakness (38.46%), and stiffness (23.08%) of the affected parts.

The other morbidities that we detected were generalized weakness (14.29%), acidity and heart burn (26.79%), menstrual problems (5.36%), insomnia (21.43%), problems with vision (12.05%), skin diseases (25%), injury (9.82%), anemia (8.93%), angular stomatitis (14.29%), pedal edema (7.14%), hypertension (16.07%), malnutrition (37.50%), swelling of feet, cough and cold, loose motion, fever, and pain abdomen.

Musculoskeletal morbidity was more common among older (>45 years) workers than in younger (<45 years) ones (77.27% *vs*. 67.78%), in females as compared to males (76.93% *vs*. 67.44%), in illiterate workers as compared to those who were literate (75.08% *vs*. 68.60%), and in substance abusers as compared to those who had no history of substance abuse (74.32% *vs*. 60.53%); however, none of these differences were statistically significant.

We also observed that musculoskeletal disorders were more common among those who had worked for more number of years (>10 years) (χ^2^ = 16.94; *P* = 0.0002, df = 2), worked for longer hours (>10 h/day) (χ^2^ = 12.67; *P* = 0.0018, df = 2), and in those who were engaged in cutting and sewing (χ^2^ = 6.38; P = 0.0412, df = 2). All these differences were statistically significant.

We organized two Focus Group Discussion to elicit evidence of social, family, and personal problems. Most of the workers were Muslims who had migrated from their rural ancestral homes in Bihar to this urban slum, which is the largest slum in Kolkata. Here, they were working under *tekhedaars*, (labor contractors) and were paid according to the number of finished goods. There was no fixed remuneration and when they fell sick they lost precious hours of work and therefore money. On the other hand, many workers took on extra work so that they could earn more money, even if the heavy workload was detrimental to their health.

The workers complained of low wages, long working hours, no relaxation time, and lack of cooperation of the employer because they were never allowed to go on leave even when they were sick and no financial help was given even if there was an emergency in the family. They were also very dissatisfied with the health care service; though the Urban Health Center under the All India Institute of Hygiene & Public Health (AIIH&PH), Kolkata, was available to cater to their health needs, the OPD timings of this center were inconvenient because it clashed with their working hours. Some of the workers suggested that since their homes were their place of work, health care service delivered at their homes would be of great help. Some felt that medical insurance for them and their families would be useful.

## Discussion

Apart from the home environment, the workplace is the setting where many people spend a large proportion of their time. But for many people, particularly in developing countries, the boundary between their home and workplace environments is blurred, since they often undertake agricultural or cottage industry activities within the home. A review of the textile industry finds that it is the largest manufacturing sector in India, accounting for around 20% of India's industrial output and 37% of its total exports.(5) We therefore feel that adequate importance should be given to the welfare of the millions of workers employed in this sector, especially those working from their homes for the small-scale garment industry.

Work provides income and thus contributes to an better socioeconomic condition which, in turn, is related to good health. However, the work environment exposes many workers to health hazards that may result in injuries, respiratory diseases, cancers, musculoskeletal disorders, reproductive disorders, cardiovascular diseases, mental and neurological illnesses, eye damage, and hearing loss, as well as communicable diseases.

Musculoskeletal problems were the commonest health problem detected in this study population. This may be explained by the fact that their work required them to remain in a bent position for many hours at a stretch, often in an overcrowded, ill-ventilated, and poorly illuminated room. The neck was the commonest anatomical area to be affected. Similar findings were reported by the Canadian Women's Health Network, with musculoskeletal disorders being the most common hazard in women engaged in sewing and the neck being the most commonly affected part, followed by the low back.(6) In another study done by How-Ran Guo, workers complained of musculoskeletal disorders of mainly neck, back, shoulders, hands, and wrists.(7)

In recent times, the contribution of poor environmental conditions at the workplace, poor perception of work conditions, and presence of adverse health conditions in workers has received much attention. The nature of workplaces varies and therefore the determinants of occupational injury and morbidity also varies; identification of the responsible factors in any specific work environment would help in clarifying the etiology and would also be useful for prevention and containment of occupation-related ill health.(8)

Stress at work is a growing problem for all workers, especially women. Many of the job conditions, along with the problem of balancing work and family issues, contribute to stress in the workplace.(9) According to the European Foundation's 1996 European Union-Wide Survey, women are more likely to have difficulty in taking breaks, days off, or holidays.(10) In this study it was observed that the majority of the women workers had to perform their household activities in addition to their work, and as a result of this family care was affected. Women had little time to take rest, to attend to personal health problems, and to attend to social engagements.

## Conclusion

The variety of morbidities detected among garment workers, especially the high prevalence of musculoskeletal problems, is alarming. It is high time that steps are taken for revising their wages and the other conditions related to their jobs so that they can improve their socioeconomic condition. Counseling for alcohol and tobacco addiction is necessary and they must be educated regarding the prevention of common diseases and the importance of personal hygiene.

Periods of rest in between their long hours of work and seats with adjustable backrests that provide support for the lumbar region would go a long way to reduce postural strain and low back pain. The responsibility for improving the health and safety conditions of garment workers lies with the government and nongovernmental agencies as well as the employers. We recommend that studies with larger sample size should be undertaken to confirm the findings of this study.

## References

[1] (1997). Occupational health: The work place. Health and environment in sustainable development.

[2] Srivastava AK, Bihari V (2000). Occupational health for women: A current need. J Sci Indian Res.

[3] Kaila HL (2000). Occup health of women. Indian J Occup Health.

[4] (1998). ILO Encyclopedia. Occupational Health and Safety.

[5] Prakash S (1998). Trade and development case studies, Country Studies, India, Part 4: Textiles [monograph on the Internet].

[6] A Call to Action. Women's Health at Work and Musculoskeletal Disorders. The Canadian Women's Health Network.

[7] Guo HR, Chang YC, Yeh WY, Chen CW, Guo YL (2004). Prevalence of musculoskeletal disorder among workers in Taiwan: A nationwide study. J Occup Health.

[8] Saha A, Nag A, Nag PK (2006). Occupational injury proneness in Indian women: A survey in fish processing industries. J Occup Med Toxicol.

[9] (2001). NIOSH looks for women's safety and health at work. Editorial.

[10] Women's health and safety, Hazards magagine.

